# Long non-coding RNA PAARH promotes hepatocellular carcinoma progression and angiogenesis via upregulating HOTTIP and activating HIF-1α/VEGF signaling

**DOI:** 10.1038/s41419-022-04505-5

**Published:** 2022-02-02

**Authors:** Huamei Wei, Zuoming Xu, Liucui Chen, Qing Wei, Zihua Huang, Guoman Liu, Wenchuan Li, Jianchu Wang, Qianli Tang, Jian Pu

**Affiliations:** 1 Department of Pathology, Affiliated Hospital of Youjiang Medical University for Nationalities, Baise, China; 2 Department of Hepatobiliary Surgery, Affiliated Hospital of Youjiang Medical University for Nationalities, Baise, China; 3 Graduate College of Youjiang Medical University for Nationalities, Baise, China

**Keywords:** Cancer microenvironment, Oncogenes, Translational research

## Abstract

Hepatocellular carcinoma (HCC) is one of the leading lethal malignancies and a hypervascular tumor. Although some long non-coding RNAs (lncRNAs) have been revealed to be involved in HCC. The contributions of lncRNAs to HCC progression and angiogenesis are still largely unknown. In this study, we identified a HCC-related lncRNA, CMB9-22P13.1, which was highly expressed and correlated with advanced stage, vascular invasion, and poor survival in HCC. We named this lncRNA Progression and Angiogenesis Associated RNA in HCC (PAARH). Gain- and loss-of function assays revealed that PAARH facilitated HCC cellular growth, migration, and invasion, repressed HCC cellular apoptosis, and promoted HCC tumor growth and angiogenesis in vivo. PAARH functioned as a competing endogenous RNA to upregulate HOTTIP via sponging miR-6760-5p, miR-6512-3p, miR-1298-5p, miR-6720-5p, miR-4516, and miR-6782-5p. The expression of PAARH was significantly positively associated with HOTTIP in HCC tissues. Functional rescue assays verified that HOTTIP was a critical mediator of the roles of PAARH in modulating HCC cellular growth, apoptosis, migration, and invasion. Furthermore, PAARH was found to physically bind hypoxia inducible factor-1 subunit alpha (HIF-1α), facilitate the recruitment of HIF-1α to *VEGF* promoter, and activate *VEGF* expression under hypoxia, which was responsible for the roles of PAARH in promoting angiogenesis. The expression of PAARH was positively associated with VEGF expression and microvessel density in HCC tissues. In conclusion, these findings demonstrated that PAARH promoted HCC progression and angiogenesis via upregulating HOTTIP and activating HIF-1α/VEGF signaling. PAARH represents a potential prognostic biomarker and therapeutic target for HCC.

## Introduction

Hepatocellular carcinoma (HCC), the major primary liver cancer, is one of the most common malignancies and leading cause of cancer-related death [1]. Although great advances have been made in clinical interventions against HCC, including surgical resection, hepatic transplantation, transcatheter arterial chemoembolization, and molecular targeted therapies, the prognosis of HCC is still very poor with 5-year survival of 18% [2–5]. Thus, further revealing the molecular alterations involved in HCC initiation and progression would be beneficial for providing therapeutic targets for HCC [6, 7].

Except for the malignant phenotypes of HCC cells, tumor microenvironment (TME) also plays critical roles in promoting HCC development [8, 9]. Hypoxia is one of the critical characteristics of most solid cancers, including HCC [10–13]. Hypoxia inducible factor-1α (HIF-1α) is stabilized by hypoxia [14]. HIF-1α activates the expression of its targeted genes, such as *VEGF*, which induces angiogenesis [15, 16]. Angiogenesis is well-known to be involved in HCC [17, 18]. HIF-1α/VEGF signaling axis-induced angiogenesis has also been revealed in HCC [14, 19]. Anti-angiogenesis antibody ramucirumab has been approved by US FDA as a second line treatment for advanced HCC patients, supporting the crucial roles of angiogenesis in HCC. However, the molecular mechanisms underlying the angiogenesis of HCC are still largely unknown.

Increasing evidences in transcriptome sequencings have identified more and more non-coding transcripts [20–22]. Among these transcripts, long non-coding RNAs (lncRNAs) are a class of regulatory non-coding RNAs with more than 200 nucleotides in length [23–25]. lncRNAs have been revealed to play various roles in multiple pathophysiological processes, including HCC [26–32]. For instance, lncRNA-ATB promotes the invasion-metastasis cascade in HCC [33]. lncRNA GPC3-AS1 promotes HCC cellular proliferation and migration [34]. lncRNA ANCR promotes HCC metastasis [35]. The molecular mechanisms of lncRNAs are also complex and various [36, 37]. One of the important mechanisms of cytoplasmic lncRNAs is to competitively bind microRNAs (miRNAs) [38, 39]. miRNAs are another class of regulatory non-coding RNAs with about 19-25 nucleotides in length. miRNAs are well-known to inhibit their target genes expression [40, 41]. lncRNAs relieve the repressive roles of miRNAs on their targets. These lncRNAs were also classed as competing endogenous RNA (ceRNA). For instance, lncRNA-ATB upregulated ZEB1 and ZEB2 through competitively binding miR-200 family [33]. lncRNA HNF1A-AS1 upregulated PIK3CD via competitively binding miRNA-30b-3p [38]. Another mechanism of action of lncRNAs is to physiology bind proteins and modulate the expression and/or function of the interacted proteins. For instance, GPC3-AS1 physically binds PCAF, recruits PCAF to *GPC3*, and activates *GPC3* expression [34]. MRCCAT1 physically binds PRC2, recruits PRC2 to *NPR3*, and represses *NPR3* transcription [42]. Apart from the important roles of lncRNAs, aberrant expressions of lncRNAs are frequently revealed in a variety of diseases [43]. However, the potential contributions of lncRNAs to HCC TME, particular angiogenesis are still largely unclear.

In this study, we identified a novel HCC-related lncRNA CMB9-22P13.1 (gene ID: 101927789; accession number: NR_160549) via analyzing the cancer genome atlas (TCGA) liver hepatocellular carcinoma (LIHC) dataset. We further investigated the expression, roles, and mechanism of action of this lncRNA in HCC. Our findings revealed that this lncRNA not only enhanced malignant phenotypes of HCC cells, but also promoted angiogenesis. Therefore, we named this lncRNA Progression and Angiogenesis Associated RNA in HCC (PAARH).

## Materials and methods

### Bioinformatics analyses

PAARH expression in HCC and the correlations between PAARH expression and overall survival and disease-free survival of HCC patients based on the RNA sequencing data of TCGA LIHC project were analyzed using the online in silico tool Gene Expression Profiling Interactive Analysis (GEPIA) (http://gepia.cancer-pku.cn/) [44]. Gene expression correlation in HCC tissues based on TCGA LIHC dataset was calculated using GEPIA (http://gepia.cancer-pku.cn/). Coding potential of PAARH was analyzed using Coding Potential Assessment Tool (CPAT) (http://lilab.research.bcm.edu/) [45], Coding Potential Calculator (CPC) 2.0 (http://cpc2.gao-lab.org/) [46], and TestCode (https://www.bioinformatics.org/sms/testcode.html). The miRNAs binding to PAARH and HOTTIP were predicted by LncBase Predicted v.2 (http://carolina.imis.athena-innovation.gr/diana_tools/web/index.php?r=lncbasev2%2Findex-predicted) [47]. The binding between PAARH and HIF-1α was predicted by RNA-Protein Interaction Prediction (RPISeq) (http://pridb.gdcb.iastate.edu/RPISeq/index.html) [48].

### Clinical samples

Seventy-two pairs of HCC tissues and adjacent noncancerous liver tissues were acquired at the Affiliated Hospital of Youjiang Medical University for Nationalities from HCC patients who received surgical resection with written informed consents. All tissues were diagnosed by two experienced pathologists. The clinicopathological characteristics of these 72 HCC cases were shown in Supplementary Table 1. This study was undertaken following the Declaration of Helsinki and approved by the Ethics Committee of Affiliated Hospital of Youjiang Medical University for Nationalities.

### Cell cultures

Human HCC cell lines SNU-398 and SK-HEP-1, and Human umbilical vein endothelial cell (HUVEC) were purchased from American Type Culture Collection (ATCC, Manassas, VA, USA). Human HCC cell line Huh7 was purchased from National Collection of Authenticated Cell Cultures of Chinese Academy of Sciences (Shanghai, China). SNU-398 was maintained in RPMI 1640 medium (Invitrogen, Carlsbad, CA USA) added with 10% fetal bovine serum (FBS, Invitrogen). SK-HEP-1 was maintained in Eagle’s Minimum Essential Medium (Invitrogen) added with 10% FBS. HUVEC was maintained in Vascular Cell Basal Media (ATCC) supplemented with Endothelial Cell Growth Kit (ATCC). Huh7 was maintained in Dulbecco’s modified Eagle’s medium (Invitrogen) added with 10% FBS. All cells were cultured at 37 °C containing 5% CO_2_ under normoxia. Under hypoxia, cells were cultured at 37 °C containing 1% O_2_, 94% N_2_, and 5% CO_2_. The cells were authenticated using STR profiles. All cells were routinely tested as mycoplasma-free.

### Quantitative real-time polymerase chain reaction (qRT-PCR)

Total RNA was isolated from indicated tissues and cells using TRIzol Reagent (Invitrogen). Next, the RNA was used to perform reverse transcription to generate complementary DNA (cDNA) by the M-MLV Reverse Transcriptase (Invitrogen) and random primers. The cDNA was further used to perform qRT-PCR by the TB Green Premix Ex Taq II (TaKaRa, Tokyo, Japan) on StepOnePlus Real-Time PCR System (Applied Biosystems, Foster City, CA, USA). The primer sequences were 5′-CCCTGAAGATAAGGTTGTGC-3′ (sense) and 5′-CTGTGGTTTGCTGAGGAGTT-3′ (anti-sense) for PAARH, 5′-CCTCCTCACCAAAAATGG-3′ (sense) and 5′-GGCAGGGCTGTACTCAAAT-3′ (anti-sense) for HOTTIP, 5′-AGGAGTACCCTGATGAGAT-3′ (sense) and 5′-GCCTTGGTGAGGTTTGAT-3′ (anti-sense) for VEGF, 5′-TGGAGAAATAGTAGATGGC-3′ (sense) and 5′-GGTGAGGAAGTAAAAACAG-3′ (anti-sense) for MALAT1, 5′-GTCGGAGTCAACGGATTTG-3′ (sense) and 5′-TGGGTGGAATCATATTGGAA-3′ (anti-sense) for GAPDH. GAPDH was used as an endogenous control. For the quantification of miRNAs, TaqMan Advanced miRNA Assays (Thermo Fisher, Waltham, MA, USA) were performed on StepOnePlus Real-Time PCR System following the manufacturer’ protocols. Relative quantification was calculated using the comparative Ct method.

### Plasmids construction and transfection

PAARH full-length sequences were PCR-amplified with the primers 5′-CCCAAGCTTGCACACTCTTGAAGTGACCG-3′ (sense) and 5′-CGGGATCCGAGATGGAGTCTCGCTCTTG-3′ (anti-sense). The PCR products were cloned into the Hind III and BamH I sites of pcDNA3.1(+) (Invitrogen) to generate PAARH overexpression plasmid pcDNA3.1-PAARH. Furthermore, the PCR products were also cloned into the Hind III and BamH I sites of pSPT19 (Roche, Basel, Switzerland) to generate PAARH in vitro transcription plasmid pSPT19-PAARH. Two pairs of cDNA oligonucleotides targeting PAARH were synthesized and cloned into the shRNA lentivirus expressing plasmid pLV3/H1/GFP&Puro (GenePharma, Shanghai, China). One pairs of cDNA oligonucleotides targeting HOTTIP were synthesized and cloned into pLV3/H1/GFP&Puro. The constructed plasmid was co-transfected with pGag/Pol, pRev and pVSV-G (GenePharma) into HEK-293FT cells to generate shRNA lentivirus targeting PAARH or HOTTIP. Scrambled non-targeting shRNA lentivirus were used as negative control (NC). The shRNA oligonucleotide sequences were 5′-GATCCGCAAGTCAACCCTGTACAACCTTCAAGAGAGGTTGTACAGGGTTGACTTGCTTTTTTG-3′ (sense) and 5′-AATTCAAAAAAGCAAGTCAACCCTGTACAACCTCTCTTGAAGGTTGTACAGGGTTGACTTGCG-3′ (anti-sense) for shRNA-PAARH-1, 5′-GATCCGCCTGAAGACACAGGACAATGTTCAAGAGACATTGTCCTGTGTCTTCAGGCTTTTTTG-3′ (sense) and 5′-AATTCAAAAAAGCCTGAAGACACAGGACAATGTCTCTTGAACATTGTCCTGTGTCTTCAGGCG-3′ (anti-sense) for shRNA-PAARH-2, 5′-GATCCGTCCTGACCAATGTAAGTGTCTTCAAGAGAGACACTTACATTGGTCAGGACTTTTTTG-3′ (sense) and 5′-AATTCAAAAAAGTCCTGACCAATGTAAGTGTCTCTCTTGAAGACACTTACATTGGTCAGGACG-3′ (anti-sense) for shRNA-HOTTIP, 5′-GATCCGTTCTCCGAACGTGTCACGTTTCAAGAGAACGTGACACGTTCGGAGAACTTTTTTG-3′ (sense) and 5′-AATTCAAAAAAGTTCTCCGAACGTGTCACGTTCTCTTGAAACGTGACACGTTCGGAGAACG-3′ (anti-sense) for shRNA-NC. PAARH sequences containing miR-6760-5p, miR-6512-3p, miR-1298-5p, miR-6720-5p, miR-4516, and miR-6782-5p binding sites were PCR-amplified with the primers 5′-CGCTCGAGAAGTGACCGGCCTGGAGA-3′ (sense) and 5′-GCTCTAGAGTGCAGTGGCACGATCTCG-3′ (anti-sense). The PCR products were cloned into the Xho I and Xba I sites of pmirGLO Dual-Luciferase miRNA Target Expression Vector (Promega, Madison, WI, USA) to generate pmirGLO-PAARH. HOTTIP sequences containing miR-6760-5p, miR-6512-3p, miR-1298-5p, miR-6720-5p, miR-4516, and miR-6782-5p binding sites were PCR-amplified with the primers 5′-CTAGCTAGCGTGAAAGTGGGCACATTA-3′ (sense) and 5′-CCGCTCGAGAACAAAAGAAACCAGAACAT-3′ (anti-sense), followed by being cloned into the Nhe I and Xho I sites of pmirGLO to generate pmirGLO-HOTTIP. The MS2-12× fragment was PCR-amplified from pSL-MS2-12× (Addgene, Watertown, MA, USA) with the primers 5′-ATGATATCCCGGGCCCTATATATGGATC-3′ (sense) and 5′-CCGCTCGAGTATCGATCGCGCGCAGATCTA-3′ (anti-sense) [49], followed by being cloned into the EcoR V and Xho I sites of pcDNA3.1 or pcDNA3.1-PAARH to generate pcDNA3.1-MS2-12× or pcDNA3.1-PAARH-MS2-12×. *VEGF* promoter sequences were PCR-amplified with the primers 5′-GGGGTACCTAGCACCTCCACCAAACC-3′ (sense) and 5′-CCCAAGCTTCACAGCCTGAAAATTACCC-3′ (anti-sense), followed by being cloned into the Kpn I and Hind III sites of pGL3-Basic Luciferase Reporter Vector to generate pGL3-*VEGF* promoter.

### miRNAs, siRNAs, and transfection

miR-6760-5p, miR-6512-3p, miR-1298-5p, miR-6720-5p, miR-4516, and miR-6782-5p mimics and NC were purchased from Thermo Fisher. ON-TARGETplus Human DICER1 siRNA SMARTpool was purchased from Dharmacon (Cambridge, England). The transfection and co-transfection of miRNAs, siRNAs, and plasmids were undertaken with Lipofectamine 3000 (Invitrogen).

### Stable cell lines construction

To construct PAARH stably overexpressed and control HCC cells, PAARH overexpression plasmid pcDNA3.1-PAARH or empty plasmid pcDNA3.1 was transfected into SNU-398 and SK-HEP-1 cells. Forty-eight hours after transfection, the cells were treated with 800 µg/ml neomycin for four weeks to select PAARH overexpressed cells. To construct PAARH stably silenced and control HCC cells, SUN-398 and Huh7 cells were infected with shRNA lentivirus targeting PAARH or scrambled non-targeting shRNA lentivirus. Ninety-six hours after infection, the cells were treated with 2 µg/ml puromycin for four weeks to select PAARH silenced cells. To construct PAARH overexpressed and concurrently HOTTIP depleted HCC cells, PAARH overexpressed SNU-398 cells were infected with shRNA lentivirus targeting HOTTIP. Ninety-six hours after infection, the cells were treated with 2 µg/ml puromycin and 800 µg/ml neomycin for four weeks to select PAARH overexpressed and concurrently PAARH silenced cells.

### Cell growth, apoptosis, migration, and invasion assays

Cell growth was evaluated using Cell Counting Kit-8 (CCK-8) and 5-ethynyl-2′-deoxyuridine (EdU) incorporation assays. For CCK-8 assays, 2000 indicated cells re-suspended in 100 µl media were seeded into 96-well plate. After culture for 4 days, 10 µl CCK-8 reagents (Dojindo, Kumamoto, Japan) was added to each well. After culture for another 2 h, the absorbance values at 450 nm were detected by a microplate reader (BioTek, Winooski, VT, USA) to indicate the number of viable cells. For EdU incorporation assays, indicated cells were treated with 50 μM EdU (RiboBio, Guangzhou, China) for 2 h, followed by being fixed in 4% paraformaldehyde for 30 min and permeabilized in 0.5% Triton X-100 for 10 min. Then, the cells were stained using Apollo dye solution (RiboBio) and the cell nucleuses were stained using DAPI. The number of EdU-positive cells was detected by a fluorescence microscope (Carl Zeiss, Oberkochen, Germany). Cellular growth was indicated by the ratio of EdU-positive cells to all cells. Cell apoptosis was evaluated by the Caspase-3 Activity Assay Kit (Cell Signaling Technology, Danvers, MA, USA) following the manufacturer’ protocol. Transwell migration and invasion assays were carried out to evaluate cell migration and invasion as we previously described [50].

### Mice xenograft models

The use of mice was reviewed and approved by the Animal Ethics Committee of Affiliated Hospital of Youjiang Medical University for Nationalities. Five-week-old male BALB/C athymic nude mice were obtained from Shanghai SLAC Laboratory Animal Co. and fed in Specific Pathogen Free conditions. Luciferase-labeled SNU-398 cells with PAARH overexpression or silencing were subcutaneously injected into nude mice. When the subcutaneous xenografts grew to about 1 cm in diameter, they were removed and cut into small pieces, which were then transplanted into the liver of nude mice. At the 14th day after transplantation, the tumors were detected by bioluminescence imaging using IVIS^@^ Lumina II system (Caliper Life Sciences, Hopkinton, MA, USA). No statistical method was used to determine sample size. The experiments were not randomized. The investigators performed the bioluminescence imaging were blinded to mouse allocation.

### Immunohistochemistry (IHC) and terminal deoxynucleotidyl transferase (TdT)-mediated dUTP nick end labeling (TUNEL)

The liver orthotopic xenografts were used to perform IHC staining as previously described with primary antibodies against Ki67 (#9027, 1:400, Cell Signaling Technology) or CD31 (ab182981, 1:1000, Abcam, Cambridge, MA, USA) [49]. The liver orthotopic xenografts were also used to undertake TUNEL assays with the TUNEL Cell Apoptosis Detection Kit (Beyotime, Shanghai, China). Human HCC tissues were used to carry out IHC staining with primary antibodies against CD31 (ab9498, 1 µg/ml, Abcam) as above described.

### Dual-luciferase reporter assays

miR-6760-5p, miR-6512-3p, miR-1298-5p, miR-6720-5p, miR-4516, or miR-6782-5p mimics were co-transfected with pmirGLO, pmirGLO-PAARH, or pmirGLO-HOTTIP into SNU-398 cells. Forty-eight hours after transfection, the Firefly luciferase and Renilla luciferase activities were measured using the Dual-Luciferase Reporter Assay System (Promega) following the provided protocol. pmirGLO or pmirGLO-HOTTIP was transfected into SNU-398 cells with PAARH overexpression or silencing. The luciferase activities were measured as above described 48 h after transfection. pGL3-Basic or pGL3-*VEGF* promoter was co-transfected with pRL-TK (Promega) into SNU-398 cells with PAARH overexpression or silencing. The luciferase activities were measured as above described 48 h after transfection. pRL-TK, which encodes Renilla luciferase, was used as endogenous control.

### RNA immunoprecipitation (RIP)

For MS2 based RIP assays, pcDNA3.1-MS2-12× or pcDNA3.1-PAARH-MS2-12× was co-transfected with pMS2-GFP (Addgene) into SNU-398 cells. Forty-eight hours after transfection, the cells were used to undertake RIP assays with a GFP antibody (11814460001, 5 µg per reaction, Roche) and the Magna RIP RNA-Binding Protein Immunoprecipitation Kit (Millipore) following the manufacturer’s protocol. To detect the RNAs binding to HIF-1α, SNU-398 cells were cultured in hypoxia (1% O_2_) for 24 h. Then, the cells were used to undertake RIP assays with a HIF-1α antibody (NB100-105, 5 µg per reaction, Novus, Littleton, CO, USA) and the Magna RIP RNA-Binding Protein Immunoprecipitation Kit. The enriched RNAs were measured using qRT-PCR as above described.

### RNA pull-down

PAARH was in vitro transcribed from pSPT19-PAARH using the MEGAscript^®^ Kit (Thermo Fisher) with T7 RNA polymerase. PAARH was labeled using the Pierce™ RNA 3′ End Desthiobiotinylation Kit (Thermo Scientific). SNU-398 cells were cultured in hypoxia (1% O_2_) for 24 h. Then, the cells were used to undertake RNA pull-down assays with the Pierce^™^ Magnetic RNA-Protein Pull-Down Kit (Thermo Scientific). The RNA present in the pull-down material was measured using qRT-PCR as above described, and the protein present in the pull-down material was measured by western blot.

### Western blot

Total protein present in the pull-down material was separated by sodium dodecyl sulfate-polyacrylamide gel electrophoresis, followed by being transferred onto polyvinylidene fluoride membrane. After being blocked in 5% skimmed milk, the membranes were incubated with primary antibodies against HIF-1α (NB100-105, 1:500, Novus) or GAPDH (ab8245, 1:5000, Abcam). The second antibody used was IRDye 680RD Goat anti-Mouse IgG (Li-Cor, Lincoln, NE, USA). Lastly, the membranes were scanned on an Odyssey infrared scanner (Li-Cor).

### In vitro tube formation

Indicated HCC cells were cultured in normoxia or hypoxia (1% O_2_) for 24 h. Then, the supernatant was collected to be used as conditioned medium (CM) for HUVEC. Each well of prechilled 24-well plates was coated with 100 µl Matrigel (BD Biosciences, Franklin Lakes, NJ, USA) and incubated at 37 °C for 1 h. In total, 2 × 10^4^ HUVEC re-suspended in above-described CM were seeded into the solidified gel. After culture for 16 h, the endothelial tubes were counted under photomicroscope.

### Enzyme linked immunosorbent assay (ELISA)

VEGF concentration in cell culture supernatant was measured by ELISA using the Human VEGF Quantikine ELISA Kit (R&D Systems, Minneapolis, MN, USA) following the manufacturer’s protocol. The absorbance values were measured by a microplate reader (BioTek). Serial dilution of human recombinant VEGF was performed in each assay to draw a standard curve.

### Chromatin immunoprecipitation (ChIP)

Indicated HCC cells were cultured in normoxia or hypoxia (1% O_2_) for 24 h. Then, the cells were used to carry out ChIP assays with the EZ-Magna ChIP A/G (17-10086, Millipore) and a HIF-1α antibody (NB100-105, 5 µg per reaction, Novus) following the provided protocol. The enriched DNA was measured using qRT-PCR with the primers 5′-CTGGCGGGTAGGTTTGAATC-3′ (sense) and 5′-AGAACGGGAAGCTGTGTGG-3′ (anti-sense) to detect *VEGF* promoter.

### Statistical analysis

GraphPad Prism 6.0 Software was employed to perform all statistical analyses. Wilcoxon matched-pairs signed rank test, log-rank test, Student’s *t* test, one-way ANOVA followed by Dunnett’s multiple comparisons test, Mann–Whitney test, Kruskal–Wallis test followed by Dunn’s multiple comparisons test, and Pearson chi-square test were undertaken as shown in the figure and table legends. *p* < 0.05 was considered statistically significant.

## Results

### PAARH was highly expressed and correlated with advanced stage, vascular invasion, and poor survival in HCC

**Fig. 1:**
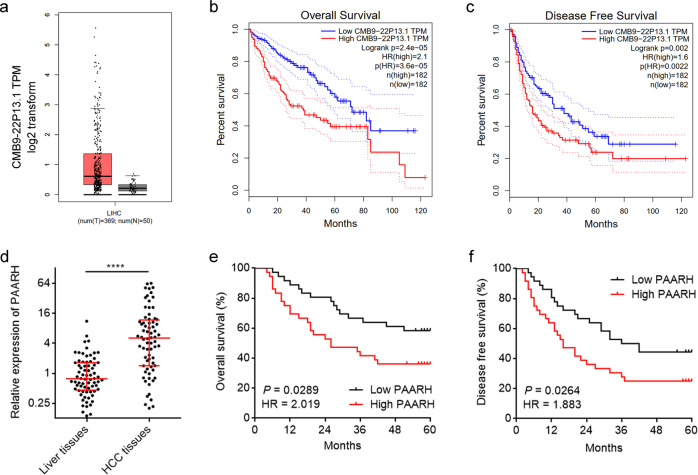
PAARH was highly expressed and correlated with poor survival in HCC. **a** PAARH (CMB9-22P13.1) expression in 369 HCC tissues and 50 liver tissues according to TCGA LIHC dataset, analyzed by GEPIA. **b** The correlation between PAARH (CMB9-22P13.1) expression and overall survival according to TCGA LIHC dataset, analyzed by GEPIA. **c** The correlation between PAARH (CMB9-22P13.1) expression and disease-free survival according to TCGA LIHC dataset, analyzed by GEPIA. **d** PAARH expression in 72 pairs of HCC tissues and adjacent noncancerous liver tissues was measured by qRT-PCR. ****p < 0.0001 by Wilcoxon matched-pairs signed rank test. **e** Kaplan–Meier survival analysis of the correlation between PAARH expression and overall survival in our HCC cohort. *n* = 72, *p* = 0.0289, HR = 2.019 by log-rank test. **f** Kaplan–Meier survival analysis of the correlation between PAARH expression and disease-free survival in our HCC cohort. *n* = 72, *p* = 0.0264, HR = 1.883 by log-rank test.

RNA-seq data of TCGA LIHC revealed that PAARH (CMB9-22P13.1) was highly expressed in HCC tissues compared with normal liver tissues (Fig. 1a), analyzed by GEPIA (http://gepia.cancer-pku.cn/index.html) [44]. RNA-seq data of TCGA LIHC also revealed that high expression of PAARH was associated with worse overall survival and disease-free survival (Fig. 1b, c). Furthermore, we randomly collected 72 pairs of HCC tissues and matched adjacent noncancerous liver tissues. qRT-PCR results confirmed the increased expression of PAARH in HCC tissues (Fig. 1d). Analyses of the correlations between PAARH expression and clinicopathological characteristics in these 72 HCC cases revealed that high PAARH expression was correlated with advanced Barcelona Clinic Liver Cancer (BCLC) stage, poor differentiation, and microvascular invasion (Supplementary Table 1). Kaplan–Meier survival analyses in these 72 HCC cases also revealed that high expression of PAARH was associated with worse overall survival and disease-free survival (Fig. 1e, f). Three in silico tools, CPAT (http://lilab.research.bcm.edu/), CPC 2.0 (http://cpc2.gao-lab.org/), and TestCode (https://www.bioinformatics.org/sms/testcode.html) all indicated PAARH as a non-coding RNA (Supplementary Fig. 1a–c). Biochemical fractionation of SNU-398 cells followed by qRT-PCR revealed that PAARH was distributed in both cytoplasm and nucleus (Supplementary Fig. 1d). These data demonstrated that PAARH was a highly expressed lncRNA in HCC. High expression of PAARH was correlated with advanced stage, poor differentiation, microvascular invasion, poor overall survival, and poor disease-free survival.

### PAARH facilitated HCC cellular growth, migration, and invasion, and inhibited HCC cellular apoptosis

**Fig. 2:**
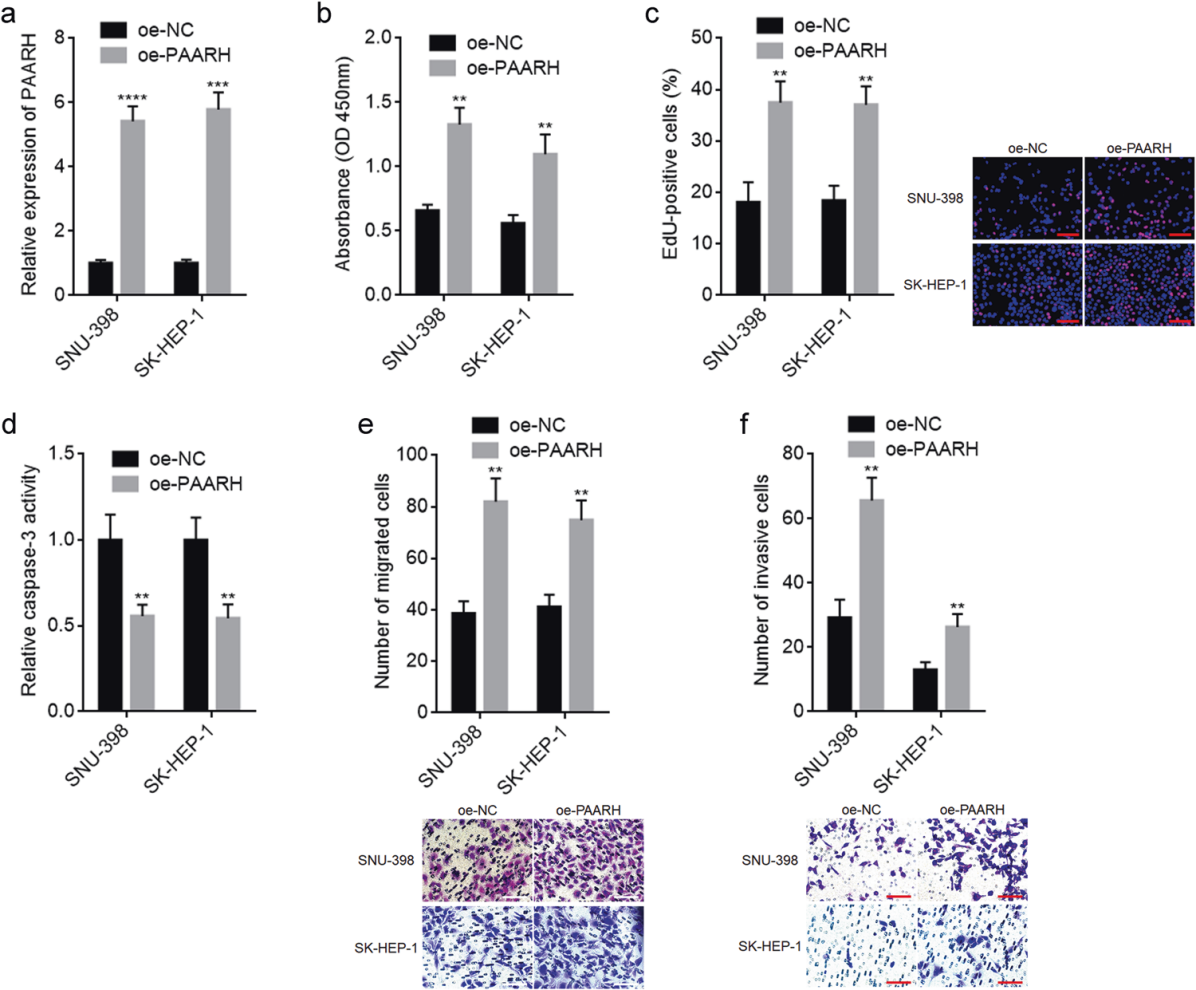
Ectopic expression of PAARH enhanced HCC cellular malignant phenotype in vitro. **a** PAARH expression in SNU-398 and SK-HEP-1 cells with PAARH stable overexpression was measured by qRT-PCR. **b** Cell growth of SNU-398 and SK-HEP-1 cells with PAARH overexpression was detected using CCK-8 assays. **c** Cell growth of SNU-398 and SK-HEP-1 cells with PAARH overexpression was detected using EdU incorporation assays. Scale bars, 100 µm. **d** Cell apoptosis of SNU-398 and SK-HEP-1 cells with PAARH overexpression was detected using caspase-3 activity assays. **e** Cell migration of SNU-398 and SK-HEP-1 cells with PAARH overexpression was detected using transwell migration assays. Scale bars, 100 µm. **f** Cell invasion of SNU-398 and SK-HEP-1 cells with PAARH overexpression was detected using transwell invasion assays. Scale bars, 100 µm. Results are shown as mean ± SD based on three independent experiments. **p < 0.01, ***p < 0.001, ****p < 0.0001 by Student’s *t* test.

Given the clinical relevance of PAARH in HCC, we further investigated the potential biological roles of PAARH in HCC. SNU-398 and SK-HEP-1 cells with PAARH stable overexpression were constructed via transfection of PAARH overexpression plasmid (Fig. 2a). CCK-8 and EdU incorporation assays presented that ectopic expression of PAARH facilitated HCC cellular growth (Fig. 2b, c). Caspase-3 activity assays presented that ectopic expression of PAARH inhibited HCC cellular apoptosis (Fig. 2d). Transwell migration and invasion experiments presented that ectopic expression of PAARH facilitated HCC cellular migration and invasion (Fig. 2e, f). These data demonstrated that ectopic expression of PAARH facilitated HCC cellular growth, migration, and invasion, and inhibited HCC cellular apoptosis.

### PAARH silencing restricted HCC cellular growth, migration, and invasion, and promoted HCC cellular apoptosis

**Fig. 3:**
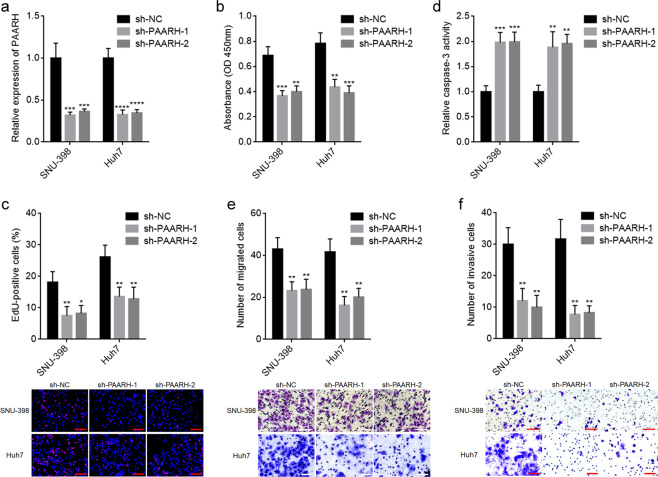
PAARH silencing reduced HCC cellular malignant phenotype in vitro. **a** PAARH expression in SNU-398 and Huh7 cells with PAARH stable silencing was measured by qRT-PCR. **b** Cell growth of SNU-398 and Huh7 cells with PAARH silencing was detected using CCK-8 assays. **c** Cell growth of SNU-398 and Huh7 cells with PAARH silencing was detected using EdU incorporation assays. Scale bars, 100 µm. **d** Cell apoptosis of SNU-398 and Huh7 cells with PAARH silencing was detected using caspase-3 activity assays. **e** Cell migration of SNU-398 and Huh7 cells with PAARH silencing was detected using transwell migration assays. Scale bars, 100 µm. **f** Cell invasion of SNU-398 and Huh7 cells with PAARH silencing was detected using transwell invasion assays. Scale bars, 100 µm. Results are shown as mean ± SD based on three independent experiments. *p < 0.05, **p < 0.01, ***p < 0.001, ****p < 0.0001 by one-way ANOVA followed by Dunnett’s multiple comparisons test.

To completely explore the potential biological roles of PAARH in HCC, SNU-398 and Huh7 cells with PAARH stable silencing were constructed via infection of two independent PAARH specific shRNA lentiviruses (Fig. 3a). CCK-8 and EdU incorporation assays presented that PAARH silencing restricted HCC cellular growth (Fig. 3b, c). Caspase-3 activity assays presented that PAARH silencing promoted HCC cellular apoptosis (Fig. 3d). Transwell migration and invasion experiments presented that PAARH silencing restricted HCC cellular migration and invasion (Fig. 3e, f). These data demonstrated that PAARH silencing restricted HCC cellular growth, migration, and invasion, and promoted HCC cellular apoptosis.

### PAARH facilitated HCC tumor progression in vivo

**Fig. 4:**
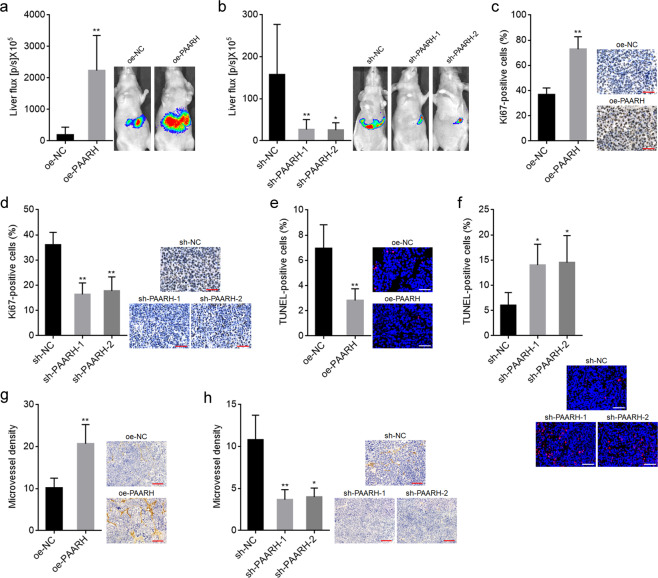
PAARH facilitated HCC progression in vivo. **a** Bioluminescence imaging of liver tumors in mice at day 14 after inoculation with small pieces of subcutaneous tumors formed by luciferase-labeled SNU-398 cells with PAARH stable overexpression or control. **b** Bioluminescence imaging of liver tumors in mice at day 14 after inoculation with small pieces of subcutaneous tumors formed by luciferase-labeled SNU-398 cells with PAARH stable silencing or control. **c** Ki67 IHC staining of liver tumors formed by SNU-398 cells with PAARH stable overexpression or control. Scale bars, 50 µm. **d** Ki67 IHC staining of liver tumors formed by SNU-398 cells with PAARH stable silencing or control. Scale bars, 50 µm. **e** TUNEL staining of liver tumors formed by SNU-398 cells with PAARH stable overexpression or control. Scale bars, 50 µm. **f** TUNEL staining of liver tumors formed by SNU-398 cells with PAARH stable silencing or control. Scale bars, 50 µm. **g** Microvessel density of liver tumors formed by SNU-398 cells with PAARH stable overexpression or control was detected using CD31 IHC staining. Scale bars, 100 µm. **h** Microvessel density of liver tumors formed by SNU-398 cells with PAARH stable silencing or control was detected using CD31 IHC staining. Scale bars, 100 µm. Results are shown as mean ± SD based on *n* = 6 mice in each group. *p < 0.05, **p < 0.01 by Mann–Whitney test (**a**, **c**, **e**, **g**) or Kruskal–Wallis test followed by Dunn’s multiple comparisons test (**b**, **d**, **f**, **h**).

To further explore the potential roles of PAARH in mouse xenograft models, small pieces of subcutaneous tumors formed by luciferase-labeled SNU-398 cells with PAARH stable overexpression or silencing were orthotopically transplanted into the livers of nude mice. Bioluminescence imaging showed that the xenografts derived from PAARH overexpressed SNU-398 cells were significantly larger than those derived from control SNU-398 cells (Fig. 4a). The xenografts derived from PAARH silenced SNU-398 cells were remarkably smaller than those derived from control SNU-398 cells (Fig. 4b). Proliferation marker Ki67 IHC staining of orthotropic xenografts revealed that the xenografts derived from PAARH overexpressed SNU-398 cells had more Ki67 positive cells than those derived from control SNU-398 cells (Fig. 4c). Conversely, the xenografts derived from PAARH silenced SNU-398 cells had less Ki67 positive cells than those derived from control SNU-398 cells (Fig. 4d). Apoptosis marker TUNEL staining of orthotropic xenografts revealed that the xenografts derived from PAARH overexpressed SNU-398 cells had less apoptotic cells than those derived from control SNU-398 cells (Fig. 4e). Conversely, the xenografts derived from PAARH silenced SNU-398 cells had more apoptotic cells than those derived from control SNU-398 cells (Fig. 4f). In addition, CD31 IHC staining orthotropic xenografts revealed that the xenografts derived from PAARH overexpressed SNU-398 cells had increased microvessel density (MVD) compared with those derived from control SNU-398 cells (Fig. 4g). Conversely, the xenografts derived from PAARH silenced SNU-398 cells had decreased MVD compared with those derived from control SNU-398 cells (Fig. 4h). These data demonstrated that PAARH also exerted oncogenic roles in vivo. PAARH not only facilitated HCC cellular malignant phenotype, but also promoted angiogenesis.

### PAARH upregulated HOTTIP via sponging miRNAs

**Fig. 5:**
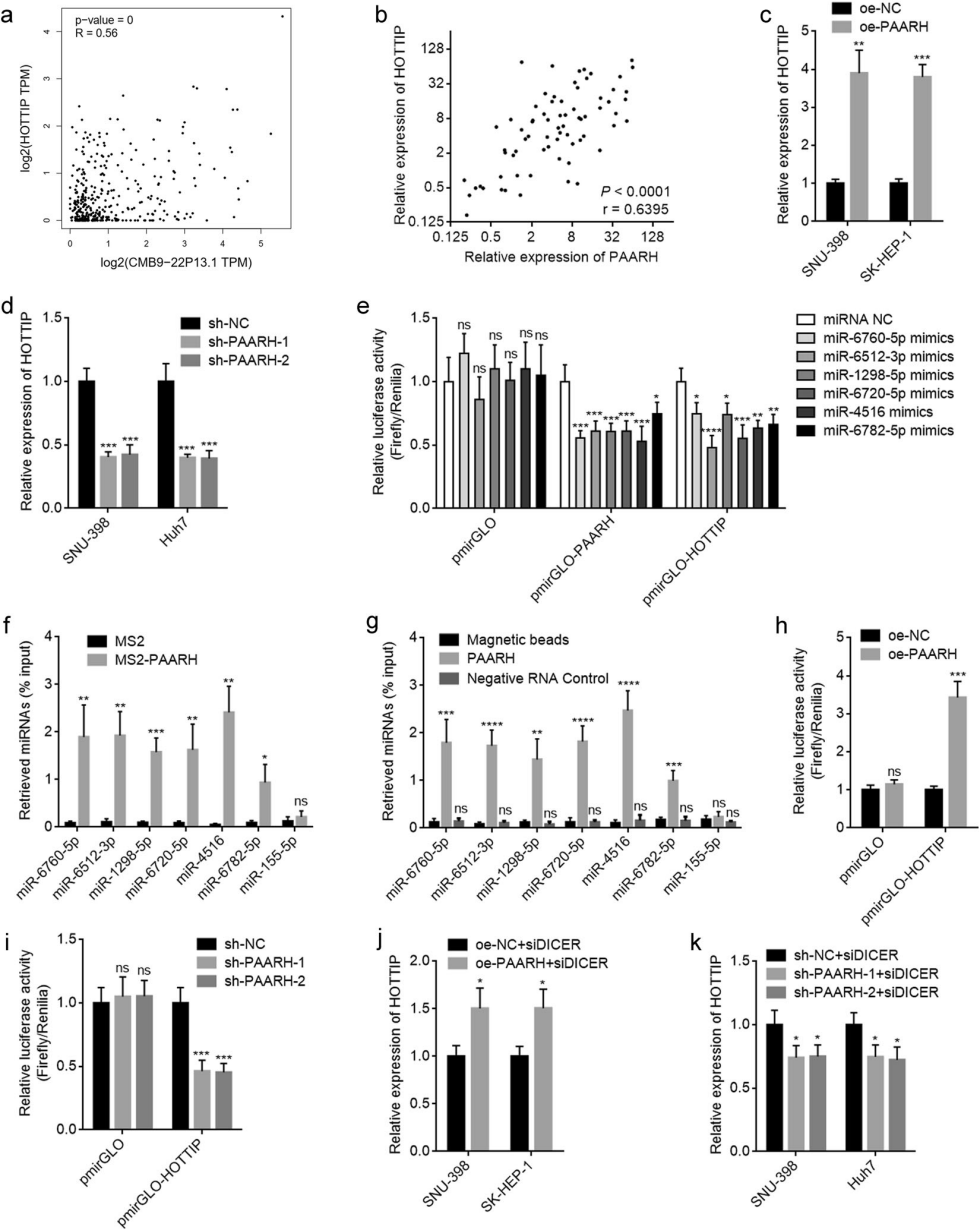
PAARH increased HOTTIP expression via functioning as a ceRNA. **a** The correlation between HOTTIP and PAARH (CMB9-22P13.1) expression in HCC tissues according to TCGA LIHC dataset, analyzed by GEPIA. **b** The correlation between HOTTIP and PAARH expression in our HCC cohort. *n* = 72, *p* < 0.0001, *r* = 0.6395 by Spearman correlation analysis. **c** HOTTIP expression levels in SNU-398 and SK-HEP-1 cells with PAARH overexpression or control were measured by qRT-PCR. **d** HOTTIP expression levels in SNU-398 and Huh7 cells with PAARH stable silencing or control was measured by qRT-PCR. **e** Luciferase activity in SNU-398 cells co-transfected with miR-6760-5p, miR-6512-3p, miR-1298-5p, miR-6720-5p, miR-4516, or miR-6782-5p and luciferase reporters containing nothing, PAARH or HOTTIP. Results are shown as the relative ratio of firefly luciferase activity to renilla luciferase activity. **f** MS2 based RIP assays followed by qRT-PCR to detect miRNAs bound to PAARH. **g** RNA pull-down experiments using RNA end-labeled with desthiobiotin, followed by qRT-PCR to detect miRNAs bound to PAARH. **h** Luciferase activity in SNU-398 cells with PAARH overexpression or control after transfection of luciferase reporters containing nothing or HOTTIP. Results are shown as the relative ratio of firefly luciferase activity to renilla luciferase activity. **i** Luciferase activity in SNU-398 cells with PAARH silencing or control after transfection of luciferase reporters containing nothing or HOTTIP. Results are shown as the relative ratio of firefly luciferase activity to renilla luciferase activity. **j** HOTTIP expression levels in SNU-398 and SK-HEP-1 cells with PAARH overexpression or control after transfection of DICER siRNA pool were measured by qRT-PCR. **k** HOTTIP expression levels in SNU-398 and Huh7 cells with PAARH stable silencing or control after transfection of DICER siRNA pool was measured by qRT-PCR. For **c**–**k**, results are shown as mean ± SD based on three independent experiments. *p < 0.05, **p < 0.01, ***p < 0.001, ****p < 0.0001, ns not significant by Student’s *t* test (**c**, **f**, **h**, **j**) or one-way ANOVA followed by Dunnett’s multiple comparisons test (**d**, **e**, **g**, **i**, **k**).

To explore the mechanisms underlying the oncogenic roles of PAARH in HCC, we searched the genes whose expressions are correlated with PAARH in HCC tissues based on TCGA LIHC dataset analyzed by GEPIA. HOTTIP ranked the second with Pearson correlation coefficient of 0.56 (Fig. 5a). The positive correlation between HOTTIP and PAARH expression was further verified in our HCC cohort (Fig. 5b). HOTTIP is a well-known oncogenic lncRNA in various malignancies, including HCC [51–53]. HOTTIP expression in PAARH overexpressed and silenced HCC cells was measured by qRT-PCR. As shown in Fig. 5c, d, HOTTIP was significantly increased in SNU-398 and SK-HEP-1 cells after PAARH overexpression, and significantly reduced in SNU-398 and Huh7 cells after PAARH silencing. Due to PAARH was distributed in both cytoplasm and nucleus. Many cytoplasmic lncRNAs were revealed to function as competing endogenous RNA (ceRNA) via sponging common miRNAs and relieving the repressive roles of miRNAs on their targets [33, 38]. To explore whether PAARH increased HOTTIP expression through the ceRNA mechanism, the miRNAs binding to PAARH and HOTTIP were predicted by LncBase Predicted v.2 (http://carolina.imis.athena-innovation.gr/diana_tools/web/index.php?r=lncbasev2%2Findex-predicted). Intriguingly, miR-6760-5p, miR-6512-3p, miR-1298-5p, miR-6720-5p, miR-4516, and miR-6782-5p were predicted to bind both PAARH and HOTTIP (Supplementary Table 2). Dual-luciferase reporter assays presented that miR-6760-5p, miR-6512-3p, miR-1298-5p, miR-6720-5p, miR-4516, and miR-6782-5p targeted both PAARH and HOTTIP (Fig. 5e). To explore whether PAARH binds these miRNAs, MS2 based RIP assays were performed as previously described [33]. The results revealed that miR-6760-5p, miR-6512-3p, miR-1298-5p, miR-6720-5p, miR-4516, and miR-6782-5p were specially enriched in MS2-PAARH group (Fig. 5f). miR-155-5p was used as NC. To further confirm the binding of these miRNAs to PAARH, RNA pull-down experiments using RNA end-labeled with desthiobiotin were undertaken. As shown in Fig. 5g, miR-6760-5p, miR-6512-3p, miR-1298-5p, miR-6720-5p, miR-4516, and miR-6782-5p, but not miR-155-5p, were specifically enriched by PAARH, but not by negative RNA control [poly(A)_25_ RNA]. These results suggested that PAARH bound miR-6760-5p, miR-6512-3p, miR-1298-5p, miR-6720-5p, miR-4516, and miR-6782-5p. Dual-luciferase reporter assays presented that conversely with these miRNAs, ectopic expression of PAARH increased the luciferase activities of the constructed reporter containing HOTTIP (Fig. 5h). PAARH silencing reduced the luciferase activities of the constructed reporter containing HOTTIP (Fig. 5i). To further investigate whether PAARH regulates HOTTIP in a miRNA-dependent manner, DICER was knocked known to repress miRNAs biogenesis. At the condition of DICER depletion, the upregulation of HOTTIP by PAARH was largely abolished (Fig. 5j, compared with Fig. 5c). Similarly, at the condition of DICER depletion, the downregulation of HOTTIP by PAARH silencing was also largely abolished (Fig. 5k, compared with Fig. 5d). Collectively, these data demonstrated that PAARH upregulated HOTTIP via sponging miR-6760-5p, miR-6512-3p, miR-1298-5p, miR-6720-5p, miR-4516, and miR-6782-5p.

### Depletion of HOTTIP largely reversed the roles of PAARH in modulating HCC cellular growth, apoptosis, migration, and invasion

**Fig. 6:**
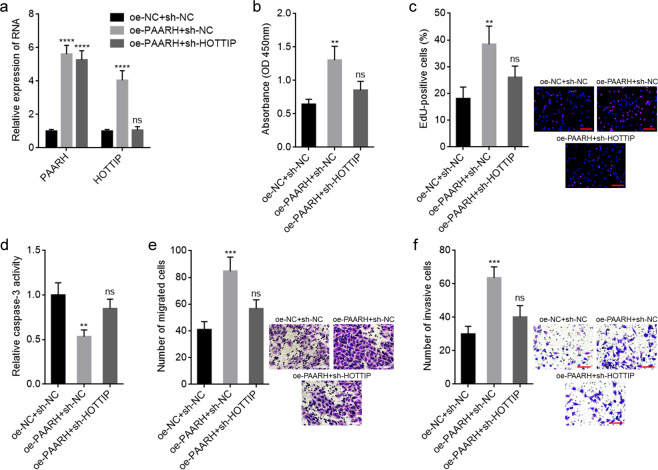
Depletion of HOTTIP reversed the oncogenic roles of PAARH in HCC. **a** PAARH and HOTTIP expression in SNU-398 cells with PAARH overexpression and concurrent HOTTIP depletion. **b** Cell growth of SNU-398 cells with PAARH overexpression and HOTTIP depletion was detected using CCK-8 assays. **c** Cell growth of SNU-398 cells with PAARH overexpression and HOTTIP depletion was detected using EdU incorporation assays. Scale bars, 100 µm. **d** Cell apoptosis of SNU-398 cells with PAARH overexpression and HOTTIP depletion was detected using caspase-3 activity assays. **e** Cell migration of SNU-398 cells with PAARH overexpression and HOTTIP depletion was detected using transwell migration assays. Scale bars, 100 µm. **f** Cell invasion of SNU-398 cells with PAARH overexpression and HOTTIP depletion was detected using transwell invasion assays. Scale bars, 100 µm. Results are shown as mean ± SD based on three independent experiments. **p < 0.01, ***p < 0.001, ****p < 0.0001, ns not significant, by one-way ANOVA followed by Dunnett’s multiple comparisons test.

Given that HOTTIP was identified as a downstream target of PAARH, we further investigated whether HOTTIP is a critical mediator of the oncogenic roles of PAARH in HCC. HOTTIP was stably depleted in PAARH overexpressed SNU-398 cells via infection of HOTTIP specific shRNA lentiviruses (Fig. 6a). CCK-8 and EdU incorporation assays presented that depletion of HOTTIP largely abolished PAARH-induced promotion of cellular growth (Fig. 6b, c). Caspase-3 activity assays presented that depletion of HOTTIP largely abolished PAARH-induced repression of apoptosis (Fig. 6d). Transwell migration and invasion experiments presented that depletion of HOTTIP largely abolished PAARH-induced promotion of migration and invasion (Fig. 6e, f). These data demonstrated that PAARH exerted oncogenic roles in HCC cellular growth, apoptosis, migration, and invasion largely through upregulating HOTTIP.

### PAARH facilitated angiogenesis via upregulating VEGF

**Fig. 7:**
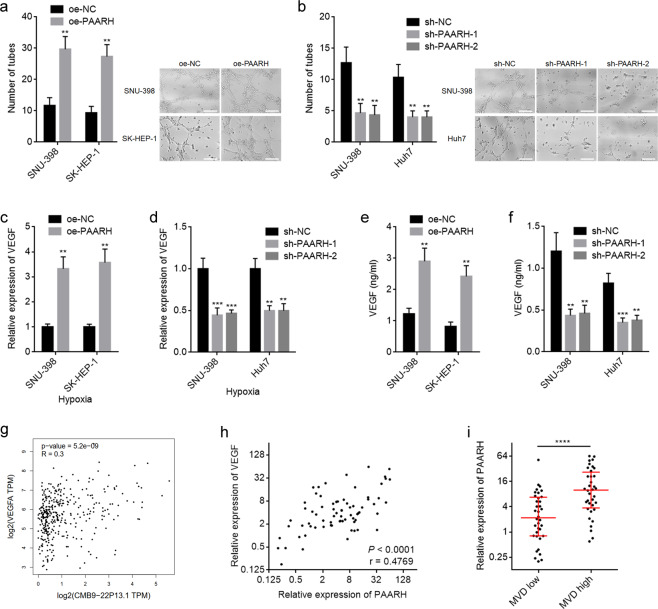
PAARH promoted angiogenesis through upregulating VEGF. **a** The number of endothelial tubes formed by HUVEC treated with conditioned medium from SNU-398 and SK-HEP-1 cells with PAARH overexpression or control under hypoxia for 24 h. Scale bars, 200 µm. **b** The number of endothelial tubes formed by HUVEC treated with conditioned medium from SNU-398 and Huh7 cells with PAARH silencing or control under hypoxia for 24 h. Scale bars, 200 µm. **c** VEGF expression in SNU-398 and SK-HEP-1 cells with PAARH overexpression or control under hypoxia for 12 h was measured by qRT-PCR. **d** VEGF expression in SNU-398 and Huh7 cells with PAARH silencing or control under hypoxia for 12 h was measured by qRT-PCR. **e** ELISA measurement of VEGF protein level in conditioned medium from SNU-398 and SK-HEP-1 cells with PAARH overexpression or control under hypoxia for 24 h. **f** ELISA measurement of VEGF protein level in conditioned medium from SNU-398 and Huh7 cells with PAARH silencing or control under hypoxia for 24 h. **g** The correlation between VEGF and PAARH (CMB9-22P13.1) expression in HCC tissues according to TCGA LIHC dataset, analyzed by GEPIA. **h** The correlation between VEGF and PAARH expression in our HCC cohort. *n* = 72, *p* < 0.0001, *r* = 0.4769 by Spearman correlation analysis. **i** PAARH expression levels in HCC tissues with high or low microvessel density (MVD), indicated by CD31 IHC staining. ****p < 0.0001 by Mann–Whitney test. For **a**–**f**, results are shown as mean ± SD based on three independent experiments. **p < 0.01, ***p < 0.001 by Student’s *t* test (**a**, **c**, **e**) or one-way ANOVA followed by Dunnett’s multiple comparisons test (**b**, **d**, **f**).

Given that PAARH expression was positively correlated with vascular invasion in HCC tissues and PAARH promoted angiogenesis in xenografts (Fig. 4g, h), we further explored the influences of PAARH on angiogenesis and the underlying mechanism. CM from PAARH overexpressed and silenced HCC cells were collected to stimulate HUVEC cells and the tube-forming abilities of HUVEC cells were detected. Under normoxia, CM from SNU-398 and SK-HEP-1 cells with PAARH overexpression did not have significant effects on tube-forming abilities of HUVEC cells (data not shown). Given that hypoxia is well-known to be involved in various malignancies, including HCC [54]. Thus, we further investigated the potential effects of PAARH on angiogenesis under hypoxia. CM from SNU-398 and SK-HEP-1 cells with PAARH overexpression significantly increased tube-forming abilities of HUVEC cells compared with CM from control SNU-398 and SK-HEP-1 cells under hypoxia (Fig. 7a). Conversely, CM from SNU-398 and Huh7 cells with PAARH silencing significantly repressed tube-forming abilities of HUVEC cells under hypoxia (Fig. 7b). VEGF is a well-known critical angiogenetic factor [55, 56]. Thus, we further investigated whether VEGF is a downstream target of PAARH in HCC. VEGF expression was significantly increased in SNU-398 and SK-HEP-1 cells after PAARH overexpression under hypoxia (Fig. 7c). Under normoxia, PAARH overexpression did not regulate VEGF (Supplementary Fig. 2a). Conversely, VEGF expression was significantly reduced in SNU-398 and Huh7 cells after PAARH silencing under hypoxia (Fig. 7d). PAARH silencing did not regulate VEGF under normoxia (Supplementary Fig. 2b). ELISA revealed that SNU-398 and SK-HEP-1 cells with PAARH overexpression had increased VEGF secretion compared with control cells under hypoxia (Fig. 7e). SNU-398 and Huh7 cells with PAARH silencing had reduced VEGF secretion compared with control cells under hypoxia (Fig. 7f). The expression of VEGF was significantly positively correlated with PAARH in HCC tissues based on TCGA LIHC dataset analyzed by GEPIA (Fig. 7g). Furthermore, the positive correlation between VEGF and PAARH expression in HCC tissues was further verified in our HCC cohort (Fig. 7h). CD31 IHC staining was performed in HCC tissues. The results revealed that PAARH was significantly upregulated in HCC tissues with high MVD compared with those with low MVD (Fig. 7i), supporting the roles of PAARH in promoting angiogenesis in vivo.

### PAARH facilitated HIF-1α-induced transcription of *VEGF* via prompting the recruitment of HIF-1α to *VEGF* promoter

**Fig. 8:**
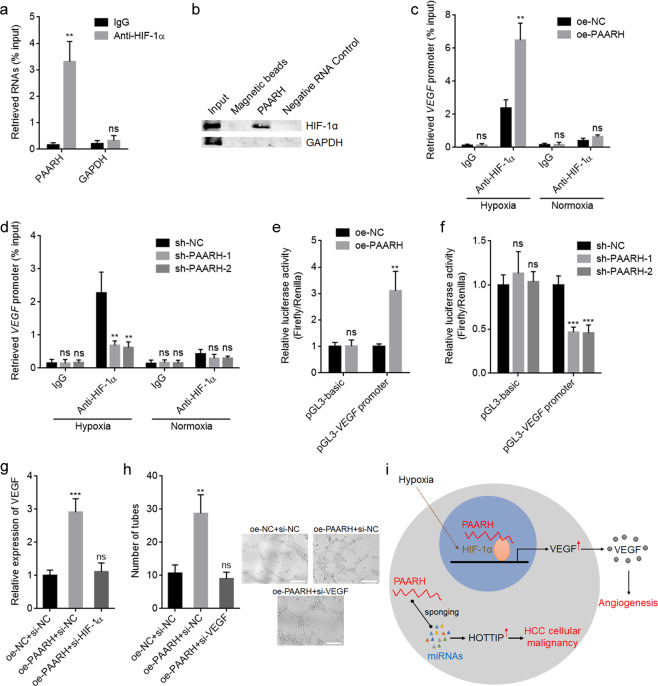
PAARH physically bound to HIF-1α, recruited HIF-1α to *VEGF* promoter, and activated *VEGF* transcription. **a** RIP assays followed by qRT-PCR to detect RNAs bound to HIF-1α. **b** RNA pull-down experiments using RNA end-labeled with desthiobiotin, followed by western blot to detect proteins bound to PAARH. **c** SNU-398 cells with PAARH overexpression or control were incubated under hypoxia or normoxia for 24 h, followed by ChIP with anti-HIF-1α antibody or nonrelated IgG. Precipitated DNAs were measured by qRT-PCR for *VEGF* promoter. **d** SNU-398 cells with PAARH silencing or control were incubated under hypoxia or normoxia for 24 h, followed by ChIP with anti-HIF-1α antibody or nonrelated IgG. Precipitated DNAs were measured by qRT-PCR for *VEGF* promoter. **e** Luciferase activity in SNU-398 cells with PAARH overexpression or control after transfection of luciferase reporters containing *VEGF* promoter or nothing. Results are shown as the relative ratio of firefly luciferase activity to renilla luciferase activity. **f** Luciferase activity in SNU-398 cells with PAARH silencing or control after transfection of luciferase reporters containing *VEGF* promoter or nothing. Results are shown as the relative ratio of firefly luciferase activity to renilla luciferase activity. **g** VEGF expression in SNU-398 cells with PAARH overexpression and concurrent HIF-1α depletion under hypoxia for 12 h was measured by qRT-PCR. **h** The number of endothelial tubes formed by HUVEC treated with conditioned medium from SNU-398 cells with PAARH overexpression and concurrent VEGF depletion under hypoxia for 24 h. Scale bars, 200 µm. **i** Schematic model of the roles of PAARH in regulating HCC cellular malignancy and angiogenesis. Results are shown as mean ± SD based on three independent experiments. **p < 0.01, ***p < 0.001, ns not significant, by Student’s *t* test (**a**, **c**, **e**) or one-way ANOVA followed by Dunnett’s multiple comparisons test (**d**, **f**–**h**).

Under hypoxia, Hypoxia Inducible Factor-1 Subunit Alpha (HIF-1α) was induced and further activated *VEGF* expression [57]. Therefore, we investigated whether HIF-1α participates in the modulation of VEGF by PAARH. PAARH was predicted to bind HIF-1α by RNA-Protein Interaction Prediction (RPISeq) (http://pridb.gdcb.iastate.edu/RPISeq/index.html). RIP assays presented that PAARH was specifically enriched in HIF-1α antibody group (Fig. 8a). RNA pull-down assays using PAARH end-labeled with desthiobiotin presented that HIF-1α was specifically enriched by PAARH, but not by negative RNA control [poly(A)_25_ RNA] (Fig. 8b). These data demonstrated that PAARH directly bound to HIF-1α. ChIP assays revealed that ectopic expression of PAARH increased the binding of HIF-1α to *VEGF* promoter under hypoxia, but not under normoxia (Fig. 8c). Consistently, PAARH silencing reduced the binding of HIF-1α to *VEGF* promoter under hypoxia, but not under normoxia (Fig. 8d). These data demonstrated that PAARH facilitated the recruitment of HIF-1α to *VEGF* promoter under hypoxia. Dual-luciferase reporter assays showed that ectopic expression of PAARH increased *VEGF* promoter activity under hypoxia (Fig. 8e). PAARH silencing decreased *VEGF* promoter activity under hypoxia (Fig. 8f). Depletion of HIF-1α abolished PAARH-induced upregulation of VEGF under hypoxia (Fig. 8g). Furthermore, Depletion of VEGF also abolished PAARH-induced increasing of tube-forming abilities of HUVEC cells under hypoxia (Fig. 8h). Collectively, these data demonstrated that PAARH physically bound HIF-1α, promoted the recruitment of HIF-1α to *VEGF* promoter, and activated *VEGF* transcription.

## Discussion

HCC is recognized as the second most lethal cancer, only after pancreatic cancer [2, 58]. The extremely poor prognosis of HCC indicated that further uncovering the molecular pathogenesis of HCC and developing more efficient systemic therapies are urgently needed.

Here, we identified a novel oncogenic lncRNA PAARH in HCC. *PAARH* is located at chromosome 11q13.1 and has three exons. Both the TCGA LIHC dataset and our own HCC cohort revealed that PAARH is highly expressed in HCC tissues compared with noncancerous liver tissues. Furthermore, both data revealed that high PAARH expression is correlated with poor overall survival and disease-free survival. Thus, PAARH is a potential prognosis biomarker for HCC, which needs further multi-central investigations. In addition, high expression of PAARH was also revealed to be positively associated with BCLC stage, poor differentiation, and microvascular invasion. These clinical relevancies implied that PAARH may exert regulatory roles in HCC.

Gain-of and loss-of function assays revealed that PAARH facilitated HCC cellular growth, migration, and invasion, and repressed HCC cellular apoptosis. In vivo xenograft assays revealed that PAARH facilitated HCC tumor growth, repressed HCC cellular apoptosis, and promoted angiogenesis. These results supported that PAARH has oncogenic roles in HCC. Intriguingly, our findings revealed that PAARH not only modulated HCC cellular malignant phenotypes in vitro and in vivo, but also modulated angiogenesis in vitro and in vivo. The important roles of PAARH in HCC suggested that targeting PAARH represents a potential therapeutic strategy against HCC, which were also verified by loss-of-function experiments in this study.

Mechanistic investigation revealed that PAARH exerted dual mechanisms (Fig. 8i). Cytoplasmic PAARH bound miR-6760-5p, miR-6512-3p, miR-1298-5p, miR-6720-5p, miR-4516, and miR-6782-5p, and relieved the repressive roles of these miRNAs on HOTTIP. Thus, PAARH functioned as a ceRNA to upregulate HOTTIP in HCC. HOTTIP is frequently reported as an oncogenic lncRNA in several cancers, including acute myeloid leukemia, head and neck squamous cell carcinoma, and HCC [52, 53, 59]. Here, functional rescue assays revealed that HOTTIP is a critical downstream mediator of the oncogenic roles of PAARH in HCC cellular malignant phenotype. The positive correlation between PAARH and HOTTIP expression in clinical HCC tissues supported the modulation of HOTTIP by PAARH.

Furthermore, nuclear PAARH was found to physically bind HIF-1α, recruited HIF-1α to *VEGF* promoter, and activated *VEGF* expression. The modulation of VEGF by PAARH is hypoxia dependent. HIF-1α protein is stabilized by hypoxia and translocated into nuclei to activate its targeted genes, such as *VEGF* [60]. Here, our findings identified HIF-1α as an interaction partner of PAARH. The binding of PAARH to HIF-1α did not change HIF-1α protein levels, but promoted the recruitment of HIF-1α to *VEGF* promoter, leading to the upregulation of *VEGF* expression. Thus, PAARH represents a novel regulator of hypoxia signaling in HCC. The positive expression between PAARH and VEGF expression, PAARH expression and microvascular density in HCC tissues supported the roles of PAARH in HCC angiogenesis. Previous reports have identified several factors regulating HIF-1α/VEGF signaling axis-induced angiogenesis in HCC, such as Cbx4 and Hepatitis C virus glycoprotein [14, 19]. In this study, we showed that lncRNA is another class of important factor regulating HIF-1α/VEGF-induced angiogenesis in HCC.

In conclusion, this study identified PAARH as a novel HCC-related lncRNA, which was frequently upregulated in HCC and associated with BCLC stage, poor differentiation, microvascular invasion, poor overall survival, and poor disease-free survival. PAARH enhanced HCC cellular malignancies via sponging miRNAs and further upregulating HOTTIP. PAARH also promoted angiogenesis via binding HIF-1α and further increasing *VEGF* expression (Fig. 8i). These data implied PAARH as a potential prognostic biomarker and therapeutic target for HCC.

## Supplementary information

agreement from all authors

Supplementary Figure legends

Supplementary Figure 1

Supplementary Figure 2

Supplementary Table 1

Supplementary Table 2

checklist

## Data Availability

The datasets generated and/or analyzed during the current study are available from the corresponding author on reasonable request.
