# Supplementary material for: Long non-coding RNA PAARH promotes hepatocellular carcinoma progression and angiogenesis via upregulating HOTTIP and activating HIF-1α/VEGF signaling
Source: Cell Death Dis. 2022 Feb 2;13(2):102. doi: 10.1038/s41419-022-04505-5 (PMC8810756; doi:10.1038/s41419-022-04505-5)
Supplement: Supplementary file 1 — agreement from all authors [file 41419_2022_4505_MOESM1_ESM.pdf]

Re:来自pu jian的邮件

weihuamei <weihuamei@yeah.net>

收件人: "pu jian" <jian_pu@126.com>

时　间: 2021-12-24 19:56:58

附　件:

I agree to these changes. Thank you.

At 2021-12-24 19:39:55, "pu jian" <jian_pu@126.com> wrote:

> Dear Huamei Wei, Zuoming Xu, Liucui Chen, Qing Wei, Zihua Huang, Guoman Liu, Wenchuan Li, Jianchu Wang, Qianli Tang, Ya Zhang, Zebang Qin, and Anmin Wang,
>
> I submitted our manuscript (CDDIS-21-3335) entitled“Long non-coding RNA PAARH promotes hepatocellular carcinoma progression andangiogenesis via upregulating HOTTIP and activating HIF-1α/VEGF signaling” to CellDeath & Disease. During the revision, considering the contributions of authors, we decided to add Qing Wei, Zihua Huang, and Guoman Liu as the authors, and delete Ya Zhang, Zebang Qin, and Anmin Wang. Editorial office of CellDeath & Disease request agreement from all authors. Please reply to this email confirming your agreement to these changes.
>
> Sincerely,
>
> Jian Pu

Re: 来自pu jian的邮件

xuzuoming@21cn.com
收件人: "pu jian" <jian_pu@126.com>
时 间: 2021-12-26 8:51:25
附 件:

Dear Mr. Pu,

I totally agree to these changes.Thank Mr. Pu.

Best regards,

Zuoming Xu

------------------ 原始邮件 ------------------

发件人: pu jian "jian_pu@126.com"
时 间: 2021/12/24 19:39:55 周五
收件人: weihuamei@yeah.net, xuzuoming@21cn.com, ChenLiucui@foxmail.com, wq_9090@163.com, 1320468196@qq.com, liuguoman221@163.com, wenchuan_li@21cn.com, Jianchu_Wang@foxmail.com, tang_qianli@yeah.net, yazhang20@aliyun.com, qinzebang@qq.com, wang_anmin@aliyun.com
抄送人:
主 题: 来自pu jian的邮件

Dear Huamei Wei, Zuoming Xu, Liucui Chen, Qing Wei, Zihua Huang, Guoman Liu, Wenchuan Li, Jianchu Wang, Qianli Tang, Ya Zhang, Zebang Qin, and Anmin Wang,

I submitted our manuscript (CDDIS-21-3335) entitled“Long non-coding RNA PAARH promotes hepatocellular carcinoma progression andangiogenesis via upregulating HOTTIP and activating HIF-1α/VEGF signaling” to CellDeath & Disease. During the revision, considering the contributions of authors, we decided to add Qing Wei, Zihua Huang, and Guoman Liu as the authors, and delete Ya Zhang, Zebang Qin, and Anmin Wang. Editorial office of CellDeath &

回复：来自pu jian的邮件

ChenLiucui <ChenLiucui@foxmail.com>

收件人："pu jian" <jian_pu@126.com>

时 间：2021-12-25 22:51:11

附 件：

Dear Mr. Pu,

I agree to these changes.

I sincerely thank you.

Best regards,

Liucui Chen

原始邮件

发件人："pu jian"< jian_pu@126.com >;

发件时间：2021/12/24 19:39

收件人："weihuamei"< weihuamei@yeah.net >;"xuzuoming"< xuzuoming@21cn.com >;"ChenLiucui"< ChenLiucui@foxmail.com >;"wq_9090"< wq_9090@163.com >;"1320468196"< 1320468196@qq.com >;"liuguoman221"< liuguoman221@163.com >;"wenchuan_li"< wenchuan_li@21cn.com >;"Jianchu_Wang"< Jianchu_Wang@foxmail.com >;"tang_qianli"< tang_qianli@yeah.net >;"yazhang20"< yazhang20@aliyun.com >;"qinzebang"< qinzebang@qq.com >;"wang_anmin"< wang_anmin@aliyun.com >;

主题：来自pu jian的邮件

Dear Huamei Wei, Zuoming Xu, Liucui Chen, Qing Wei, Zihua Huang, Guoman Liu, Wenchuan Li, Jianchu Wang, Qianli Tang, Ya Zhang, Zebang Qin, and Anmin Wang,

I submitted our manuscript (CDDIS-21-3335) entitled“Long non-coding RNA PAARH promotes hepatocellular carcinoma progression andangiogenesis via

Re:来自pu jian的邮件

MG-Qing <wq_9090@163.com>
收件人: "pu jian" <jian_pu@126.com>
时 间: 2021-12-25 20:17:59
附 件:

I totally agree.I sincerely thank Mr.Pu.
Best regards,
Qing Wei

At 2021-12-24 18:39:55, "pu jian" <jian_pu@126.com> wrote:

> Dear Huamei Wei, Zuoming Xu, Liucui Chen, Qing Wei, Zihua Huang, Guoman Liu, Wenchuan Li, Jianchu Wang, Qianli Tang, Ya Zhang, Zebang Qin, and Anmin Wang,
>
> I submitted our manuscript (CDDIS-21-3335) entitled“Long non-coding RNA PAARH promotes hepatocellular carcinoma progression andangiogenesis via upregulating HOTTIP and activating HIF-1α/VEGF signaling” to CellDeath & Disease. During the revision, considering the contributions of authors, we decided to add Qing Wei, Zihua Huang, and Guoman Liu as the authors, and delete Ya Zhang, Zebang Qin, and Anmin Wang. Editorial office of CellDeath & Disease request agreement from all authors. Please reply to this email confirming your agreement to these changes.
>
> Sincerely,
>
> Jian Pu

"ïĭ" <1320468196@qq.com>
收件人: "pu jian" <jian_pu@126.com>
时　间: 2021-12-25 20:59:34
附　件:

I totally agree.I sincerely thank Mr.Pu.

回复：来自pu jian的邮件

liuguoman221 <liuguoman221@163.com>

收件人："jian_pu@126.com" <jian_pu@126.com>

时　间：2021-12-26 9:30:00

附　件：

I totally agree. Thank Mr. Pu faithfully .

liuguoman221

liuguoman221@163.com

签名由 网易邮箱大师 定制

在2021年12月24日 19:39，pu jian<jian_pu@126.com> 写道：

Dear Huamei Wei, Zuoming Xu, Liucui Chen, Qing Wei, Zihua Huang, Guoman Liu, Wenchuan Li, Jianchu Wang, Qianli Tang, Ya Zhang, Zebang Qin, and Anmin Wang,

I submitted our manuscript (CDDIS-21-3335) entitled"Long non-coding RNA PAARH promotes hepatocellular carcinoma progression andangiogenesis via upregulating HOTTIP and activating HIF-1α/VEGF signaling" to CellDeath & Disease. During the revision, considering the contributions of authors, we decided to add Qing Wei, Zihua Huang, and Guoman Liu as the authors, and delete Ya Zhang, Zebang Qin, and Anmin Wang. Editorial office of CellDeath & Disease request agreement from all authors. Please reply to this email confirming your agreement to these changes.

Sincerely,

Jian Pu

Re: 来自pu jian的邮件

wenchuan_li@21cn.com
收件人："pu jian" <jian_pu@126.com>
时　间：2021-12-26 9:05:35
附　件：

Dear Dr. Pu,

I agree to these changes.

Thank you very much.

Best regards,

Wenchuan Li

------------------ 原始邮件 ------------------

发件人：pu jian "jian_pu@126.com"
时　间：2021/12/24 19:39:55 周五
收件人：weihuamei@yeah.net, xuzuoming@21cn.com, ChenLiucui@foxmail.com, wq_9090@163.com, 1320468196@qq.com, liuguoman221@163.com, wenchuan_li@21cn.com, Jianchu_Wang@foxmail.com, tang_qianli@yeah.net, yazhang20@aliyun.com, qinzebang@qq.com, wang_anmin@aliyun.com
抄送人：
主　题：来自pu jian的邮件

Dear Huamei Wei, Zuoming Xu, Liucui Chen, Qing Wei, Zihua Huang, Guoman Liu, Wenchuan Li, Jianchu Wang, Qianli Tang, Ya Zhang, Zebang Qin, and Anmin Wang,

I submitted our manuscript (CDDIS-21-3335) entitled“Long non-coding RNA PAARH promotes hepatocellular carcinoma progression andangiogenesis via upregulating HOTTIP and activating HIF-1α/VEGF signaling” to CellDeath & Disease. During the revision, considering the contributions of authors, we decided to add Qing Wei, Zihua Huang, and Guoman Liu as the authors, and delete Ya Zhang, Zebang Qin, and Anmin Wang. Editorial office of CellDeath & Disease request agreement from all authors. Please reply to this email confirming your agreement to these changes.

回复：来自pu jian的邮件

jianchuwang <2745699806@qq.com>

收件人："pu jian" <jian_pu@126.com>

时　间：2021-12-26 8:46:02

附　件：

OK, I agree.

原始邮件

发件人："pu jian"< jian_pu@126.com >;

发件时间：2021/12/24 19:39

收件人："weihuamei"< weihuamei@yeah.net >;"xuzuoming"< xuzuoming@21cn.com >;"ChenLiucui"< ChenLiucui@foxmail.com >;"wq_9090"< wq_9090@163.com >;"1320468196"< 1320468196@qq.com >;"liuguoman221"< liuguoman221@163.com >;"wenchuan_li"< wenchuan_li@21cn.com >;"Jianchu_Wang"< Jianchu_Wang@foxmail.com >;"tang_qianli"< tang_qianli@yeah.net >;"yazhang20"< yazhang20@aliyun.com >;"qinzebang"< qinzebang@qq.com >;"wang_anmin"< wang_anmin@aliyun.com >;

主题：来自pu jian的邮件

Dear Huamei Wei, Zuoming Xu, Liucui Chen, Qing Wei, Zihua Huang, Guoman Liu, Wenchuan Li, Jianchu Wang, Qianli Tang, Ya Zhang, Zebang Qin, and Anmin Wang,

I submitted our manuscript (CDDIS-21-3335) entitled“Long non-coding RNA PAARH promotes hepatocellular carcinoma progression andangiogenesis via upregulating HOTTIP and activating HIF-1α/VEGF signaling” to CellDeath & Disease. During the revision, considering the contributions of authors, we decided to add Qing Wei, Zihua Huang, and Guoman Liu as the authors, and

Re:来自pu jian的邮件

tang_qianli <tang_qianli@yeah.net>
收件人: "pu jian" <jian_pu@126.com>
时　间: 2021-12-25 22:32:53
附　件:

I agree. Thank you.
Qianli Tang

在 2021-12-24 19:39:55，"pu jian" <jian_pu@126.com> 写道:

> Dear Huamei Wei, Zuoming Xu, Liucui Chen, Qing Wei, Zihua Huang, Guoman Liu, Wenchuan Li, Jianchu Wang, Qianli Tang, Ya Zhang, Zebang Qin, and Anmin Wang,
>
> I submitted our manuscript (CDDIS-21-3335) entitled“Long non-coding RNA PAARH promotes hepatocellular carcinoma progression andangiogenesis via upregulating HOTTIP and activating HIF-1α/VEGF signaling” to CellDeath & Disease. During the revision, considering the contributions of authors, we decided to add Qing Wei, Zihua Huang, and Guoman Liu as the authors, and delete Ya Zhang, Zebang Qin, and Anmin Wang. Editorial office of CellDeath & Disease request agreement from all authors. Please reply to this email confirming your agreement to these changes.
>
> Sincerely,
>
> Jian Pu

回复：来自pu jian的邮件

yazhang20 <yazhang20@aliyun.com>

收件人："pu jian" <jian_pu@126.com>

时　间：2021-12-25 22:42:57

附　件:

I totally aggree to these changes.
Thank Mr. Pu.
Sincerely,
Ya Zhang

发件人：pu jian <jian_pu@126.com>
发送时间：2021年12月24日(星期五) 19:40
收件人：weihuamei <weihuamei@yeah.net>; xuzuoming <xuzuoming@21cn.com>; ChenLiucui <ChenLiucui@foxmail.com>; wq_9090 <wq_9090@163.com>; 1320468196 <1320468196@qq.com>; liuguoman221 <liuguoman221@163.com>; wenchuan_li <wenchuan_li@21cn.com>; Jianchu_Wang <Jianchu_Wang@foxmail.com>; tang_qianli <tang_qianli@yeah.net>; yazhang20 <yazhang20@aliyun.com>; qinzebang <qinzebang@qq.com>; wang_anmin <wang_anmin@aliyun.com>
主　题：来自pu jian的邮件

Dear Huamei Wei, Zuoming Xu, Liucui Chen, Qing Wei, Zihua Huang, Guoman Liu, Wenchuan Li, Jianchu Wang, Qianli Tang, Ya Zhang, Zebang Qin, and Anmin Wang,

I submitted our manuscript (CDDIS-21-3335) entitled“Long non-coding RNA PAARH promotes hepatocellular carcinoma progression andangiogenesis via upregulating HOTTIP and activating HIF-1α/VEGF signaling” to CellDeath & Disease. During the revision, considering the contributions of authors, we decided to add Qing Wei, Zihua Huang, and Guoman Liu as the authors, and delete Ya Zhang, Zebang Qin, and Anmin Wang. Editorial office of CellDeath & Disease request agreement from all authors. Please reply to this email confirming your agreement to these changes.

Sincerely,

回复：来自pu jian的邮件

"zebang qin" <536981513@qq.com>
收件人："pu jian" <jian_pu@126.com>
时 间：2021-12-26 10:05:49
附 件：

I agree to these changes. Thanks faithfully.

原始邮件

发件人："pu jian"< jian_pu@126.com >;

发件时间：2021/12/24 19:39

收件人："weihuamei"< weihuamei@yeah.net >;"xuzuoming"< xuzuoming@21cn.com >;"ChenLiucui"< ChenLiucui@foxmail.com >;"wq_9090"< wq_9090@163.com >;"1320468196"< 1320468196@qq.com >;"liuguoman221"< liuguoman221@163.com >;"wenchuan_li"< wenchuan_li@21cn.com >;"Jianchu_Wang"< Jianchu_Wang@foxmail.com >;"tang_qianli"< tang_qianli@yeah.net >;"yazhang20"< yazhang20@aliyun.com >;"qinzebang"< qinzebang@qq.com >;"wang_anmin"< wang_anmin@aliyun.com >;

主题：来自pu jian的邮件

Dear Huamei Wei, Zuoming Xu, Liucui Chen, Qing Wei, Zihua Huang, Guoman Liu, Wenchuan Li, Jianchu Wang, Qianli Tang, Ya Zhang, Zebang Qin, and Anmin Wang,

I submitted our manuscript (CDDIS-21-3335) entitled“Long non-coding RNA PAARH promotes hepatocellular carcinoma progression andangiogenesis via

回复：来自pu jian的邮件

wang_anmin <wang_anmin@aliyun.com>

收件人："pu jian" <jian_pu@126.com>

时　间：2021-12-25 22:46:51

附　件：

Dear Dr. Pu,

I totally agree. Thank you.

Best regards,

Anmin Wang

发件人：pu jian <jian_pu@126.com>

发送时间：2021年12月24日(星期五) 19:40

收件人：weihuamei <weihuamei@yeah.net>; xuzuoming <xuzuoming@21cn.com>; ChenLiucui <ChenLiucui@foxmail.com>; wq_9090 <wq_9090@163.com>; 1320468196 <1320468196@qq.com>; liuguoman221 <liuguoman221@163.com>; wenchuan_li <wenchuan_li@21cn.com>; Jianchu_Wang <Jianchu_Wang@foxmail.com>; tang_qianli <tang_qianli@yeah.net>; yazhang20 <yazhang20@aliyun.com>; qinzebang <qinzebang@qq.com>; wang_anmin <wang_anmin@aliyun.com>

主　题：来自pu jian的邮件

Dear Huamei Wei, Zuoming Xu, Liucui Chen, Qing Wei, Zihua Huang, Guoman Liu, Wenchuan Li, Jianchu Wang, Qianli Tang, Ya Zhang, Zebang Qin, and Anmin Wang,

I submitted our manuscript (CDDIS-21-3335) entitled“Long non-coding RNA PAARH promotes hepatocellular carcinoma progression andangiogenesis via upregulating HOTTIP and activating HIF-1α/VEGF signaling” to CellDeath & Disease. During the revision, considering the contributions of authors, we decided to add Qing Wei, Zihua Huang, and Guoman Liu as the authors, and delete Ya Zhang, Zebang Qin, and Anmin Wang. Editorial office of CellDeath & Disease request agreement from all authors. Please reply to this email confirming your agreement to these changes.

Sincerely,

Jian Pu
