# Supplementary material for: Long non-coding RNA PAARH promotes hepatocellular carcinoma progression and angiogenesis via upregulating HOTTIP and activating HIF-1α/VEGF signaling
Source: Cell Death Dis. 2022 Feb 2;13(2):102. doi: 10.1038/s41419-022-04505-5 (PMC8810756; doi:10.1038/s41419-022-04505-5)
Supplement: Supplementary file 2 — Supplementary Figure legends [file 41419_2022_4505_MOESM2_ESM.docx]

**Supplementary figure legends**

**Supplementary Fig. 1 The coding potential and subcellular distribution of PAARH in HCC cells. a** The coding potential of PAARH was calculated using Coding Potential Assessment Tool (CPAT). **b** The coding potential of PAARH was calculated using Coding Potential Calculator (CPC) 2.0. **c** The coding potential of PAARH was calculated using TestCode. **d** Biochemical fractionation of SNU-398 cells, followed by qRT-PCR to detect the subcellular distribution of PAARH. GAPDH and MALAT1 were used as cytoplasmic and nuclear controls, respectively.

**Supplementary Fig. 2 PAARH did not regulate VEGF under normoxia. a** VEGF expression in SNU-398 and SK-HEP-1 cells with PAARH overexpression or control under normoxia was measured by qRT-PCR. **b** VEGF expression in SNU-398 and Huh7 cells with PAARH silencing or control under normoxia was measured by qRT-PCR. Results are shown as mean ± SD based on three independent experiments. ns, not significant, by Student’s *t*-test (**a**) or one-way ANOVA followed by Dunnett's multiple comparisons test (**b**).
