# Supplementary figures and images for: Long non-coding RNA PAARH promotes hepatocellular carcinoma progression and angiogenesis via upregulating HOTTIP and activating HIF-1α/VEGF signaling
Source: Cell Death Dis. 2022 Feb 2;13(2):102. doi: 10.1038/s41419-022-04505-5 (PMC8810756; doi:10.1038/s41419-022-04505-5)

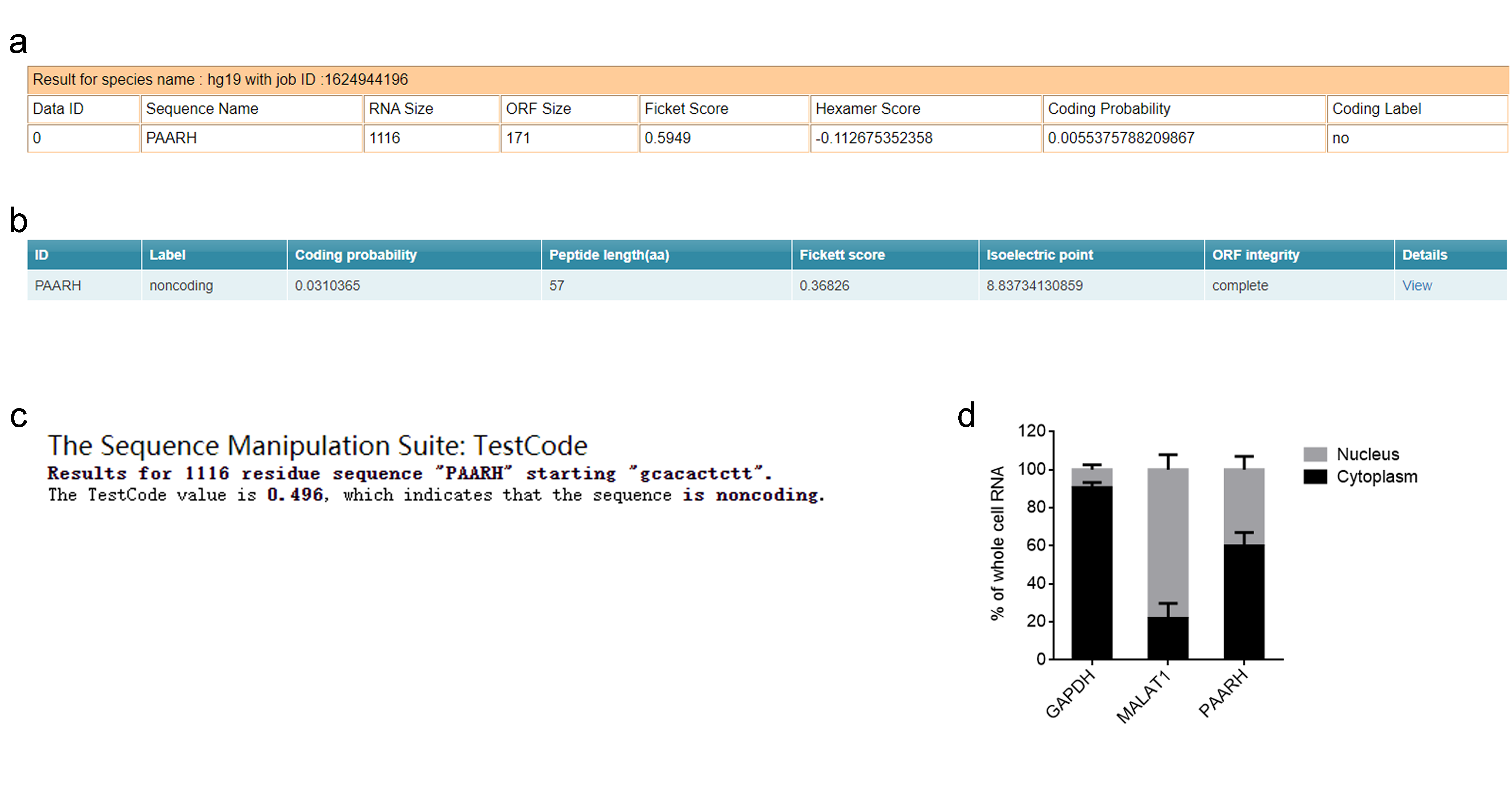

Supplement: Supplementary file 3 — Supplementary Figure 1 [file 41419_2022_4505_MOESM3_ESM.tif]

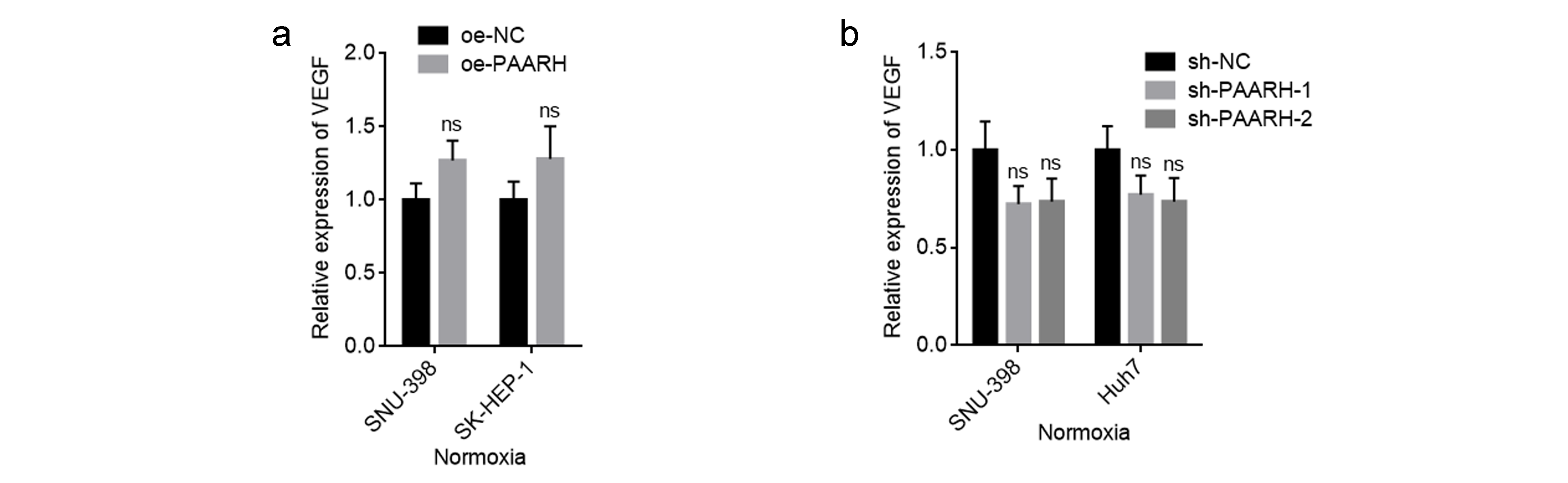

Supplement: Supplementary file 4 — Supplementary Figure 2 [file 41419_2022_4505_MOESM4_ESM.tif]
