# Supplementary material for: Long non-coding RNA PAARH promotes hepatocellular carcinoma progression and angiogenesis via upregulating HOTTIP and activating HIF-1α/VEGF signaling
Source: Cell Death Dis. 2022 Feb 2;13(2):102. doi: 10.1038/s41419-022-04505-5 (PMC8810756; doi:10.1038/s41419-022-04505-5)
Supplement: Supplementary file 5 — Supplementary Table 1 [file 41419_2022_4505_MOESM5_ESM.docx]

**Supplementary Table 1 Correlation between PAARH expression and clinicopathological characteristics in HCC.**

| Feature | PAARH | | χ^2^ | *P* value |
| --- | --- | --- | --- | --- |
|  | low | high |  |  |
| Age |  |  | 2.057 | 0.151 |
| >50 | 18 | 24 |  |  |
| ≤50 | 18 | 12 |  |  |
| Gender |  |  | 0.141 | 0.708 |
| Male | 31 | 33 |  |  |
| Female | 5 | 3 |  |  |
| HBs antigen |  |  | 0.758 | 0.384 |
| Positive | 27 | 30 |  |  |
| Negative | 9 | 6 |  |  |
| Liver cirrhosis |  |  | 0.262 | 0.609 |
| With | 12 | 10 |  |  |
| Without | 24 | 26 |  |  |
| BCLC stage |  |  | 6.020 | **0.014** |
| 0-A | 28 | 18 |  |  |
| B-C | 8 | 18 |  |  |
| AFP (ng/ml) |  |  | 2.057 | 0.151 |
| >20 | 18 | 24 |  |  |
| ≤20 | 18 | 12 |  |  |
| Differentiation |  |  | 4.181 | **0.041** |
| I-II | 8 | 2 |  |  |
| III-IV | 28 | 34 |  |  |
| Encapsulation |  |  | 3.057 | 0.217 |
| Complete | 6 | 3 |  |  |
| Not complete | 23 | 20 |  |  |
| No | 7 | 13 |  |  |
| Microvascular invasion |  |  | 11.200 | **0.001** |
| Absent | 28 | 14 |  |  |
| Present | 8 | 22 |  |  |

*P* value was acquired by Pearson chi-square test.
