# Supplementary material for: Long non-coding RNA PAARH promotes hepatocellular carcinoma progression and angiogenesis via upregulating HOTTIP and activating HIF-1α/VEGF signaling
Source: Cell Death Dis. 2022 Feb 2;13(2):102. doi: 10.1038/s41419-022-04505-5 (PMC8810756; doi:10.1038/s41419-022-04505-5)
Supplement: Supplementary file 6 — Supplementary Table 2 [file 41419_2022_4505_MOESM6_ESM.docx]

**Supplementary Table 2 The predicted miRNAs bound by PAARH and HOTTIP.**

| Transcript | Binding Position | microRNAs | Binding Category |
| --- | --- | --- | --- |
| PAARH | 91-113 | miR-6760-5p | 8mer |
| PAARH | 491-509 | miR-6760-5p | 8mer |
| PAARH | 43-64 | miR-6760-5p | 6mer |
| PAARH | 438-463 | miR-6512-3p | 9mer |
| PAARH | 148-169 | miR-6512-3p | 6mer |
| PAARH | 660-685 | miR-6512-3p | 6mer |
| PAARH | 363-387 | miR-1298-5p | 8mer |
| PAARH | 98-124 | miR-1298-5p | 7mer |
| PAARH | 640-662 | miR-1298-5p | 6mer |
| PAARH | 449-463 | miR-6720-5p | 9mer |
| PAARH | 143-169 | miR-6720-5p | 6mer |
| PAARH | 661-685 | miR-6720-5p | 6mer |
| PAARH | 491-508 | miR-4516 | 8mer |
| PAARH | 95-112 | miR-4516 | 7mer |
| PAARH | 517-537 | miR-4516 | 8mer |
| PAARH | 313-339 | miR-6782-5p | 7mer |
| HOTTIP | 1373-1397 | miR-6760-5p | 9mer |
| HOTTIP | 2197-2223 | miR-6760-5p | 6mer |
| HOTTIP | 1576-1596 | miR-6512-3p | 7mer |
| HOTTIP | 2456-2480 | miR-6512-3p | 7mer |
| HOTTIP | 3789-3806 | miR-6512-3p | 6mer |
| HOTTIP | 4203-4219 | miR-6512-3p | 6mer |
| HOTTIP | 2529-2550 | miR-6512-3p | 8mer |
| HOTTIP | 2585-2608 | miR-6512-3p | 6mer |
| HOTTIP | 4614-4636 | miR-1298-5p | 7mer |
| HOTTIP | 4161-4177 | miR-1298-5p | 6mer |
| HOTTIP | 1579-1596 | miR-6720-5p | 7mer |
| HOTTIP | 2457-2480 | miR-6720-5p | 7mer |
| HOTTIP | 3784-3806 | miR-6720-5p | 6mer |
| HOTTIP | 4194-4219 | miR-6720-5p | 6mer |
| HOTTIP | 2529-2550 | miR-6720-5p | 8mer |
| HOTTIP | 2586-2608 | miR-6720-5p | 6mer |
| HOTTIP | 1376-1395 | miR-4516 | 9mer |
| HOTTIP | 4460-4477 | miR-4516 | 8mer |
| HOTTIP | 2280-2297 | miR-4516 | 6mer |
| HOTTIP | 3982-4006 | miR-6782-5p | 7mer |
| HOTTIP | 3952-3978 | miR-6782-5p | 7mer |
| HOTTIP | 4583-4604 | miR-6782-5p | 6mer |
| HOTTIP | 3083-3107 | miR-6782-5p | 6mer |
| HOTTIP | 3350-3370 | miR-6782-5p | 6mer |
